# Morphology Distribution in Injection Molded Parts

**DOI:** 10.3390/polym16030337

**Published:** 2024-01-26

**Authors:** Sara Liparoti, Rita Salomone, Vito Speranza, Roberto Pantani

**Affiliations:** Department of Industrial Engineering, University of Salerno, Via Giovanni Paolo II, 132, 84084 Fisciano, SA, Italy; rsalomone@unisa.it (R.S.); vsperanza@unisa.it (V.S.); rpantani@unisa.it (R.P.)

**Keywords:** injection molding, annealing, mold temperature, polypropylene, mechanical performances, atomic force microscopy, orientation

## Abstract

A more sustainable use of plastic parts makes it necessary to replace current plastic parts with recyclable components, also allowing the modulation of the part properties through the process. Injection molding is one of the most widely used technologies for obtaining rigid plastic parts, so it is crucial to understand how to tailor properties by adopting the correct processing conditions. One way is to perform annealing steps directly inside the mold: in-mold annealing improves the structural integrity and durability of the material, reduces defects, increases the resistance of parts against certain chemicals, reduces wear and tear, increases ductility, and lowers brittleness. In this work, several in-mold annealing steps were conducted, changing the mold temperature and annealing duration selected on the basis of the half crystallization time of the adopted isotactic polypropylene. The typical molded part morphology, composed of oriented layers at the surface, transition zones, and spherulitic core, is strongly affected by in-mold annealing. In particular, the thickness of the oriented layer, which forms in the early phase of the process, decreases, and the spherulites increase in size. Concerning mechanical behavior, the orientation degree mostly determines the elastic modulus value close to the surface, whereas the conditions under which crystallization occurs determine the modulus in the core.

## 1. Introduction

Plastics are crucial in our economy and daily lives; for instance, they significantly reduce fossil fuel consumption in the transportation sector and improve our safety in several fields [1]. However, the large amount of plastics introduced every year is a threat to the environment and human health. Many efforts are devoted to mitigating the side effects of plastics; for instance, the Plastic Strategy of the European Union developed an action plan focused on transforming how plastic products are designed, produced, used, and recycled [2,3]. The huge amount of polymer types, often in the form of multilayers, and the presence of filler to enhance barrier and mechanical properties limit the recyclability, particularly in regions where waste collection is poorly organized [4]. Using monomaterials instead of multilayers and compounds certainly enhances the recyclability of plastics. However, utilizing monomaterials necessitates tailoring the properties of the components through the manufacturing process. 

Isotactic polypropylene is one of the most used polymers in transportation due to its low density, which allows for the production of lightweight materials [5,6]. The possibility to control the properties of isotactic polypropylene (iPP) parts in the injection molding process can be a way to reduce the environmental impact of plastic. It is known from the literature that the mechanical properties of injection molded iPP depend on the crystalline structure and lamellae orientation, with oriented structures showing higher mechanical performances [7]. The kind of semicrystalline structures formed during injection molding of non-nucleated iPP depends on the local crystallization conditions (temperature and flow field experienced by the polymer chains). This determines the overall performance of the parts [8,9,10]. Thus, tailoring the morphological structure development during injection molding would imply tailoring the final part properties. Annealing is one of the most common and effective heat treatments to improve crystallinity and reduce defects due to residual stress. It is usually performed at an intermediate temperature between glass transition and melting temperature to promote chain mobility toward a more thermodynamically stable state, modifying the final structure and enhancing the properties of the part [11,12]. During the annealing process, microstructural changes may occur, including the formation of new crystallites and the perfection of the existing ones; the form and size of crystals, as well as crystallinity, can change, significantly influencing the overall properties of the molded part. The chain mobility due to annealing induces the amorphous phase to rearrange into a crystalline phase. Moreover, for semicrystalline polymers, the crystallinity increases with crystal perfection due to secondary crystallization [11,13,14,15]. 

Most work analyzing the effects of annealing on the injection molded part properties concerns slow crystallizing polymers. Li et al. [16] found that the tensile strength, tensile modulus, and storage modulus increased with annealing time and annealing temperature, while the tensile toughness decreased in poly (lactic acid) molded parts. Boruvka et al. [17] processed PLA blends by injection molding under different mold temperatures followed by in-mold annealing. In-mold annealing increased impact toughness and tensile modulus, thanks to the increase in the crystalline degree. Dar et al. [18] investigated the influence of mold and melt temperatures on the behavior of optical-grade polycarbonate in terms of mechanical and failure properties. They adopted an annealing step in an air-circulated oven to the injection molded parts. They found that the yield stress increased, whereas strain at failure decreased with the increase in the annealing temperature and time. 

A few works focus on the effect of the annealing steps on fast-crystallizing polymers. Mi et al. [19] conducted heat treatments at several temperatures, below, near, and above the melting temperature of a PP to obtain an oriented shish-kebab structure and isolated spherulites. They found that annealing temperatures near or above the melting temperature improved the mechanical properties. There was also an increase in the crystallinity degree and lamellar thickness, which means an increase in the yield and impact strength of the spherulitic structures. Pan et al. [20] analyzed the relationship between the microstructure and mechanical properties of annealed injection molded iPP with high orientation. They observed an improvement in the mechanical properties of the annealed parts. Such an improvement was ascribed to an increase in the overall crystallinity and the recombination of the crystalline phase. Annealing induces chain rearrangement in the crystalline structures [21] with subsequent effects on the mechanical properties, such as decreased yield strength, reduced strain for the onset of strain hardening, and improved impact toughness. Wang et al. [22] found that injection molded high-density polyethylene was influenced by the annealing treatment at different temperatures. When annealing is performed from a low temperature to a high-temperature regime, other effects are shown in terms of crystallinity, lamellar thickness, and increasing yield stress. A relationship between morphology and mechanical properties through the annealing process was also observed for iPP/HDPE blends [23]. Variations of crystallinity and the crystalline structure influenced mechanical properties such as the impact strength and tensile strength. In the cases of iPP/β nucleating agents/polyethylene oxide blends, the annealing process changed the degree of orientation and improved the fracture toughness [24].

The analysis of the state of the art of the annealing process coupled with injection molding reveals that the annealing steps were mostly conducted in a separate air oven where the deformation of the parts may occur due to stress relaxation. This work explores the influence of in-mold annealing on the morphology, molecular orientation, and mechanical property distribution of an iPP. For this purpose, injection molding tests were conducted with different heating cycles to assess the effect of the mold temperature and heating duration on the part’s properties. 

## 2. Materials and Methods

The injection molding tests were carried out with an iPP (T30G, Basell, Ferrara, Italy) that had previously been characterized with regard to crystallization and rheology [25]. Tests were performed by a Haake Minijet injection molding machine (Thermo Scientific HAAKE MARS, Milan, Italy) in a rectangular cavity (60 mm length, 10 mm wide, and 1 mm thickness), adopting a 473 K injection temperature, 20 MPa injection pressure, 1 s injection time, and 4 s packing time. Different mold temperatures were selected for the injection molding tests: 25 °C, 140 °C, and 160 °C. After injection, in-mold annealing steps were conducted at different annealing temperatures and times. In particular, the mold was kept at high temperatures (140 °C or 160 °C, the same adopted during the injection molding process) for 300 s, then cooled in a water bath. In selected cases, a second annealing step was conducted at 130 °C for 2 h. The conditions of the second annealing step were chosen according to the crystallization time of the selected iPP [25]. Table 1 shows all of the operative conditions adopted during the injection molding tests.

Injection molded parts were characterized concerning their morphology and mechanical properties. The morphology was characterized by optical and electronic microscopy. In particular, thin slices (100 μm thick) were cut using a Leica microtome (RM2255, Leica Biosystems, Buccinasco, Milan, Italy) and analyzed by optical microscopy under polarized light (mod. BX51, Olympus Italia S.r.l., Segrate, Milan, Italy). For each specimen, the micrograph was taken with the slices rotated at 45° with respect to the polarizer direction. The change in brightness during a 45° rotation is generally proportional to the molecular orientation of the polymer.

Specimens cut from the central part of the slab, etched according to Basset’s procedure [26], and coated with a thin gold layer were analyzed by a desktop Scanning Electron Microscope (SEM, Phenom ProX, Phenom-World BV, Eindhoven, The Netherlands).

**Figure 1:**
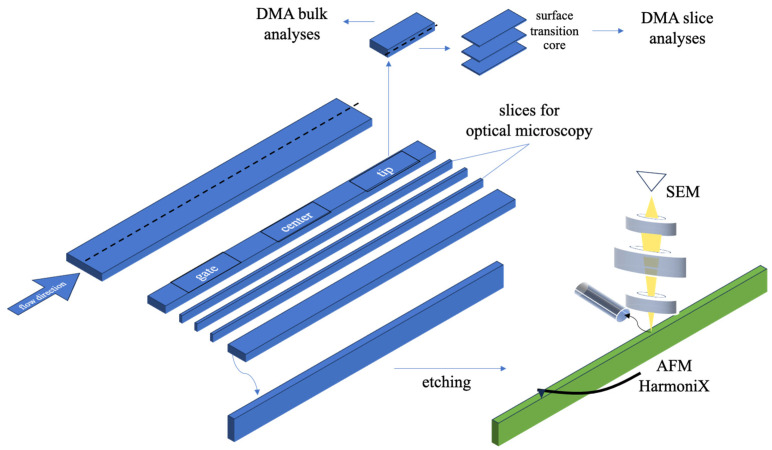
Cutting procedure adopted for the morphological and mechanical analyses.

Figure 1 shows the cutting procedure adopted for mechanical and morphological analyses. Specimens were cut at the gate position (10 mm from the cavity entrance), center (30 mm from the cavity entrance), and tip (55 mm from the cavity entrance) in different directions with respect to the flow path to obtain slices for microscopy and Dynamic Mechanical Analyzer (DMA) analyses. Specimens were treated for Atomic Force Microscopy (AFM) and SEM. Slices and specimens were cut under ambient conditions; an etching procedure [26] was applied to remove the residues formed due to the cutting procedure.

Mechanical characterizations were conducted at different levels, from the macro to the micro scale. A DMA 8000 (Perkin Elmer, Milan, Italy) was used to analyze the mechanical characteristics in dynamic and static modes. In the case of dynamic mode, the tension mode was used. A preliminary strain sweep test was conducted to assess the linear viscoelastic response of the material; this allowed us to set a 50 μm displacement and 1 Hz frequency for the tensile tests. Tests were conducted on slices (50 µm thickness) cut at different distances through the flow thickness plane (from the sample skin to the core) and on slices cut along the flow thickness plane in the orthogonal direction with respect to the skin (see the sketch in Figure 1). Concerning the static analysis, the stress–strain mode was used to characterize the bulk mechanical behavior of specimens cut at the gate, center, and tip positions; 9 N was selected as the maximum load, with a 1 N/min load rate.

Mechanical tests at the micrometrical level were conducted by an Atomic Force Microscope (AFM, NanoScope MultiMode V, Veeco, Santa Barbara, CA, USA) equipped with a HarmoniX tool. HMX probe silicon cantilevers (Bruker, Billerica, MA, USA) with nominal radii of c.a. 10 nm were adopted. AFM HarmoniX was calibrated following the procedure reported in [27], adopting a polyethylene/polystyrene standard for the calibration procedure; the AFM elastic modulus distribution was obtained by averaging the elastic moduli on windows of 20 × 20 μm.

The orientation distribution along the part thickness was characterized by the Fiji plug-in Directionality (http://fiji.sc/Fiji, accessed on 3 January 2024, Ashburn, VA, USA), following the procedure described by Liu [28]. This technique was previously applied to AFM micrographs [29]. In this work, it is used for the first time in optical micrographs. In particular, square areas with selected dimensions (depending on the structure that had to be analyzed) underwent Fast Fourier Transformation, enabling the Directionality plug-in to calculate the spatial frequencies within an image given a set of radial directions. The method generated normalized histograms, revealing the number of structures oriented along a certain direction between 0 and 180° with a bin size of 1°. In the cases proposed in this work, 90° corresponds to the flow direction during the injection molding process. The reconstruction of the signal obtained by Directionality analysis combines Lorentzian and Gaussian functions (GL(f)), where f is the direction. The direction distribution is fitted by the function GL(f).

The procedure to obtain a parameter, named d, is given by the following equation:

(1) $$d=\frac{\int_{0}^{\pi }GL(f){(\cos f)}^{2}\sin fdf}{\int_{0}^{\pi }GL(f)\sin fdf}$$

The parameter D is given by the equation $D=\frac{3d-1}{2}$.

If D close to 1, the structures are oriented along the flow direction; if D is close to 0, the structures are unoriented.

## 3. Moldflow Simulations

The injection molding process was simulated with Moldflow software (Moldflow 2018, Autodesk Inc., San Rafael, CA, USA) according to the conditions reported in Table 1 to obtain spherulite dimensions. A non-Newtonian viscous flow was considered in the simulation with material rheology described by the Cross-WLF model [30]. The existing Moldflow database material for T30G was modified according to the characterization of the iPP adopted in this work [25]. In particular, the descriptions of rheology and crystallinity were updated. The rectangular cavity was discretized in Moldflow considering the dual domain mesh that allows the evaluation of crystallinity. The mesh adopted for the simulations is composed of 12,354 triangular elements with a global edge length of 0.52 mm and 6179 nodes [30]. A previous study assessed the validity of the database material and the adopted mesh by comparing the simulations to experimental findings [25,31].

Quiescent crystallization kinetics was also used in the simulations. The nucleation density can be described by

(2) $$N=N_{0}+N_{f}$$

where $N_{0}\;$ is the number of nuclei that form during quiescent crystallization and $N_{f}$ is the number of nuclei induced by flow. In this work, the effect of flow on nucleation was not accounted for. Concerning the quiescent nucleation density, it is described by Equation (3)

(3) $$\ln N_{0}=a_{n}∆T+b_{n}$$

where $a_{n}$ and $b_{n}$ are constants of the considered iPP, and $∆T=T_{m}^{0}-T$, with $T_{m}^{0}$ the grade-specific equilibrium melting temperature, assumed to depend on pressure, following the expression $T_{m}^{0}=T_{eql}+b_{6}P$, where $b_{6}$ is a material constant.

The crystal growth rate depends on temperature and follows the Hoffman–Lauritzen theory:

(4) $$G\left(T\right)=G_{0}exp\left[-\frac{U^{*}}{R_{g}(T-T_{\infty )}}\right]exp\left[-\frac{gK_{g}}{T(T_{m}^{0}-T)}\right]$$

where $T_{\infty }=T_{g}-30$ ($T_{g}$ is the glass transition temperature), and

(5) $$g=\frac{T+T_{m}^{0}}{2T}$$

$G_{0}$ and $K_{g}$ are material grade-specific constants, which can be determined under quiescent conditions, $U^{*}$ is the activation energy of motion, and $R_{g}$ is the gas constant. Table 2 reports the values of the adopted constants.

## 4. Results

**Figure 2:**
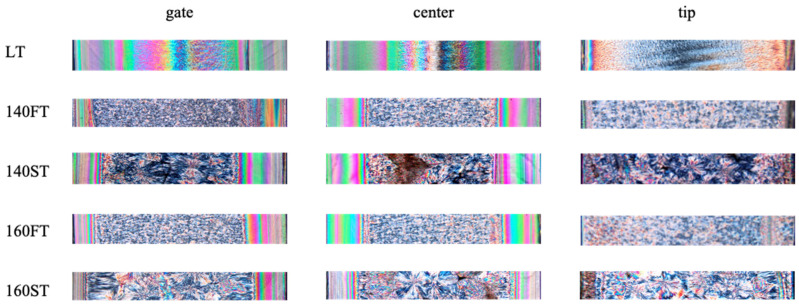
Morphology of the injection molding parts obtained in three positions along the flow path (gate, center, and tip) for samples obtained adopting several injection molding conditions, as indicated in the figure (see Table 1).

Figure 2 shows optical micrographs obtained at three positions along the flow path (see Table 1 for the operating conditions and Figure 1 for the positions along the flow path).

Parts are characterized by colored bands close to the sample surface due to the presence of an oriented layer and a grainy region in the core. The part produced at a molding temperature of 25 °C consists mainly of the oriented layer (Figure 2). Oriented layer thickness decreases with the increase in the mold temperature, and the grainy region enlarges. The enhancement of the relaxation phenomena and the delay of the crystallization, both due to the high temperatures, can explain the oriented layer reduction. The second annealing at 130 °C has a negligible effect on the oriented layer thickness because the oriented layer formation occurs during the process’s early stages.

Conversely, the second annealing seems to affect the grainy region: an increase in the spherulitic mean diameter is detectable when the second annealing step is performed. Under the second annealing conditions, the crystallization in the spherulitic region occurs under almost quiescent conditions, and the nucleation rate is low. Consequently, a few nuclei form and the spherulites are allowed to grow to a greater extent before impingement. For the samples obtained with a single annealing step, crystallization occurs during the cooling stage, when the nucleation rate is high; in these cases, more nuclei form, and impingement occurs earlier.

The sample center shows a morphological distribution similar to that found at the gate, with an increase in symmetry probably due to better temperature control in this part of the mold.

At the tip position, the morphology is mainly composed of spherulites. In this region, the effect of flow on nucleation and growth rates is small, and the formation of oriented regions is essentially hindered. A small, oriented layer is detectable only for LT samples.

**Figure 3:**
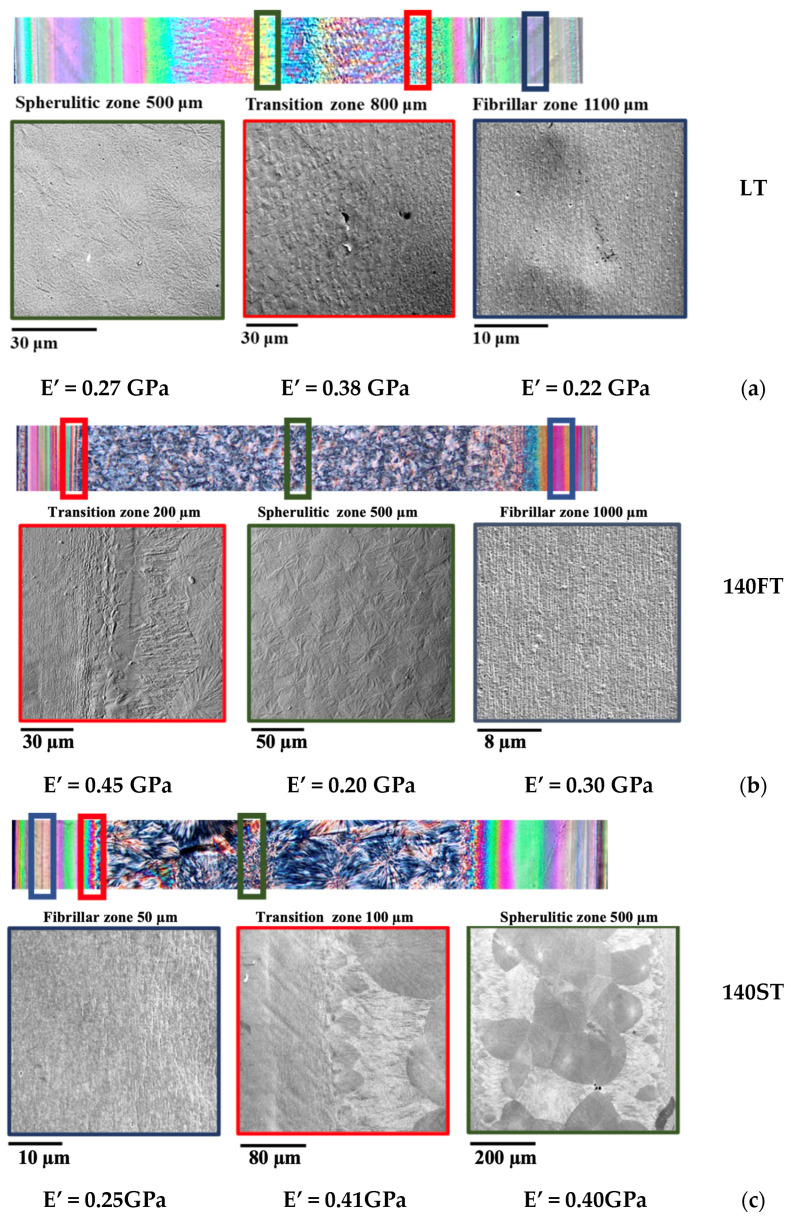

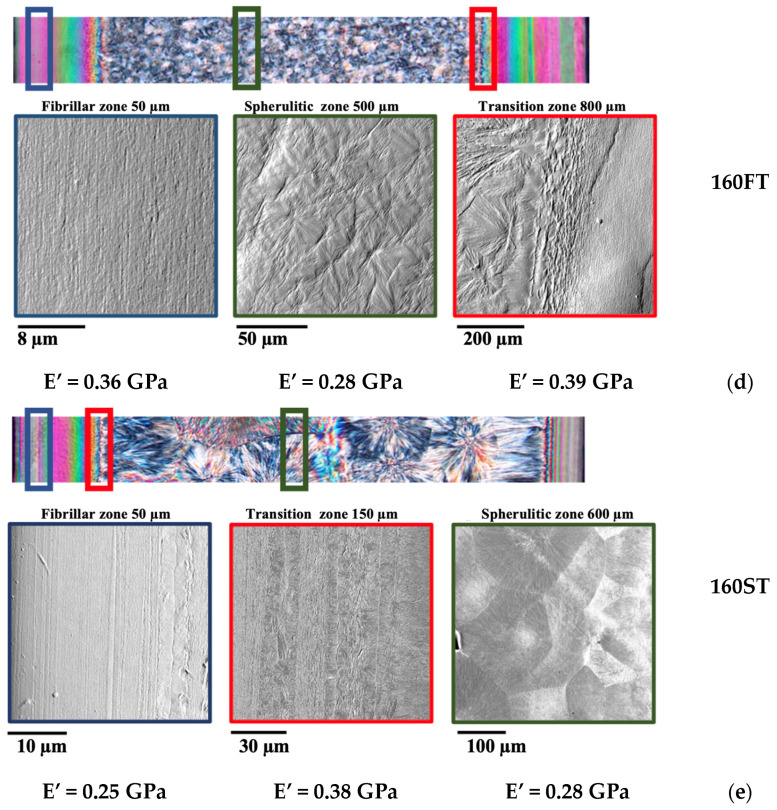
Morphology detected by optical microscopy and SEM of the injection molding parts in three positions along the thickness for samples obtained at (**a**) 25 °C mold temperature (LT); (**b**) 140 °C mold temperature maintained for 300 s (140FT); (**c**) 140 °C mold temperature maintained for 300 s followed by in-mold annealing at 130 °C, 2 h (140ST); (**d**) 160 °C mold temperature maintained for 300 s (160FT); (**e**) 160 °C mold temperature maintained for 300 s followed by in-mold annealing at 130 °C, 2 h (160ST). The distance from the sample surface is also indicated for each SEM micrograph. The moduli indicated below the pictures were measured by DMA at 30 °C. The green contour is used for the spherulitic region, the red one for the transition zone, and the blue one for the fibrillar oriented layer.

SEM micrographs allow us to characterize features in oriented and grainy regions and their dimensions. Figure 3 shows SEM micrographs captured in several positions along the sample thickness (the optical micrographs are also reported).

SEM micrographs of the regions near the walls show tightly packed fibrillar structures. Towards the sample core, a transition area can be detected, characterized by oriented structures with a morphology intermediate between fibrils and spherulites. In particular, larger and more spaced fibrils are detected, and some spherulites form with dimensions smaller than those detected toward the core. SEM micrographs confirm that the spherulites that formed in the transition region grow toward the core, whereas the growth toward the fibrillar layer is hindered. The samples are characterized by spherulites in the grainy region, whose dimensions depend on the process conditions. The DMA analyses evidenced that the transition zone presents higher modulus values.

**Figure 4:**
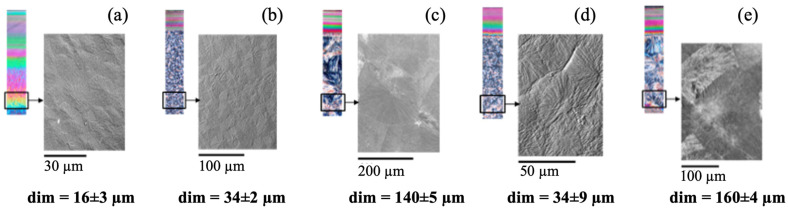
SEM micrographs in the spherulitic region for sample LT (**a**), 140FT (**b**), 140ST (**c**), 160FT (**d**), 160ST (**e**). The spherulitic mean dimension (dim) and optical micrographs are also reported for each sample.

Figure 4 shows the enlargement of the SEM micrographs in the grainy region; the average dimension of the spherulites is also reported.

The samples obtained with 25 °C mold temperature are characterized by the lowest value of the spherulitic dimensions. The increase in mold temperature and the performing of the annealing step induce an enlargement in the spherulite dimensions.

The annealing step conducted at 130 °C for 2 h induces a significant increase in the spherulite dimensions, consistent with the decrease in the nucleation rate in that temperature range; this effect confirms that the nucleation and growth in the sample core mainly proceed after the filling stage, with almost quiescent conditions. Thus, a small number of nuclei form and grow, achieving dimensions significantly larger than those generally observed in LT molding parts; this affects the mechanical performance of the part.

**Figure 5:**
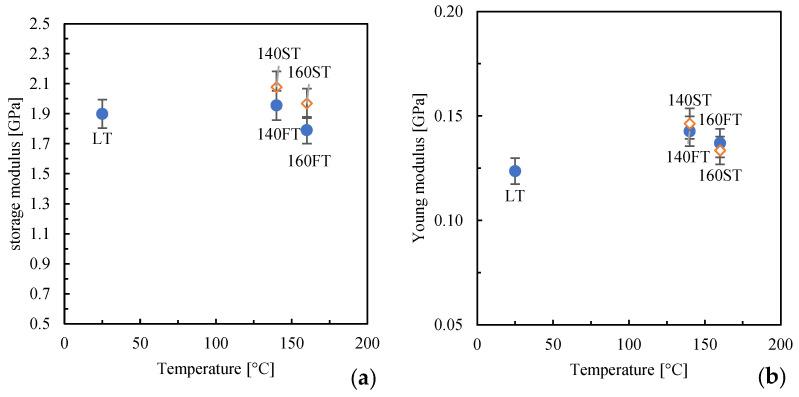
Modulus obtained from DMA analyses conducted in tension mode (**a**) and under stress–strain (**b**).

Figure 5 shows the Young and storage moduli obtained from the DMA analyses in stress–strain and tension modes. In tension mode, the storage modulus pictured in the figure relates to data acquired at low test temperatures (30 °C).

The parts obtained with high mold temperatures (140FT and 160FT) exhibit modulus values close to the LT part. When the second annealing is performed, 140ST and 160ST, a slight increase in the moduli can be observed; this finding may be ascribed to a better structuring of the molecules due to the permanence within high-temperature ranges.

**Figure 6:**
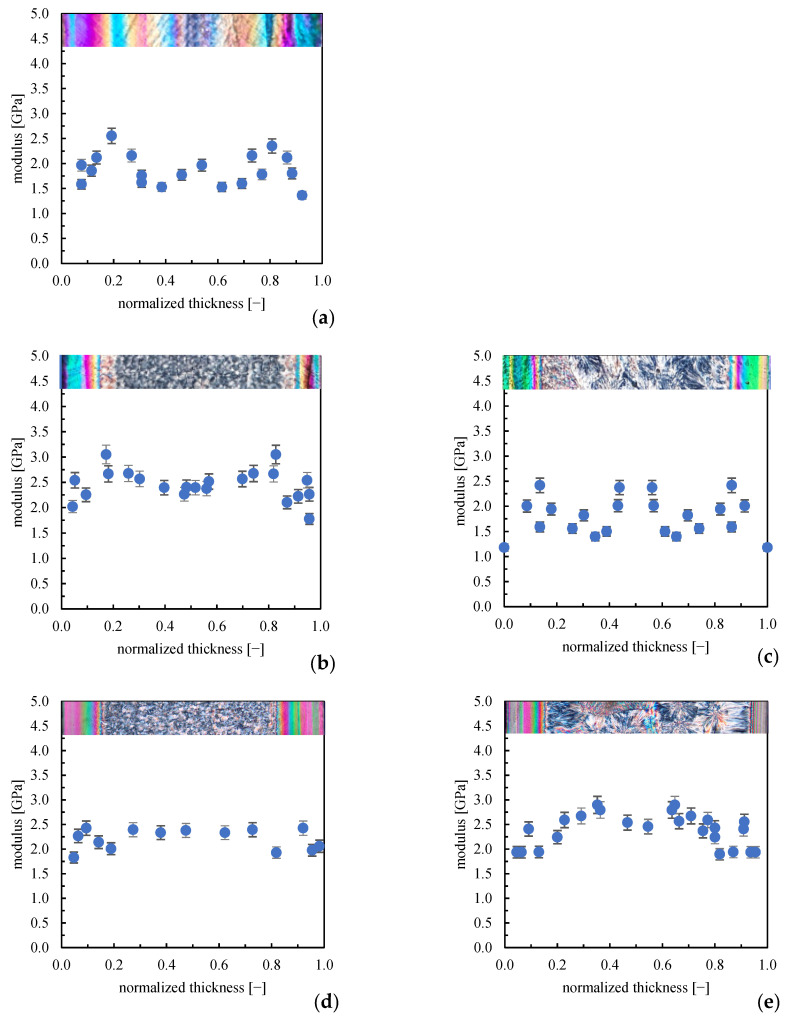
Elastic modulus distribution along the sample thickness close to the gate obtained by AFM HarmoniX acquisitions for samples (**a**) LT; (**b**) 140FT; (**c**) 140ST; (**d**) 160FT; (**e**) 160ST.

Since the process induces a different level of structuring in the parts, it can be expected that the modulus would show a certain distribution along the thickness. Figure 6 shows the elastic modulus distribution along the sample thickness in the position closest to the gate (see sketch in Figure 1).

The mold temperature and annealing influence the morphology and mechanical properties. In the LT cases (25 °C mold temperature), the high elastic modulus value is detectable in the fibrillar layer, the lowest elastic moduli characterize the transition zone, and intermediate elastic modulus values characterize the spherulitic region. For the cases with 140 and 160 °C mold temperatures (140FT and 160ST) and with the annealing steps (140ST and 160ST), the highest values of the elastic modulus move towards the sample surface, consistent with the reduction of the oriented layer thickness observed in the optical micrographs. Interestingly, the elastic modulus distribution becomes more homogenous with the increase in mold temperature; indeed, samples 160FT and 160ST show only a negligible difference between the modulus values in the core and the oriented layer.

In the core, the elastic modulus increases when the annealing is performed. The literature proved that the dimension of spherulites affects the elastic modulus: the larger the spherulite dimension, the higher the modulus [32]. Furthermore, the central part of the spherulite shows a higher modulus, due most likely to the fine and complex microstructure.

**Figure 7:**
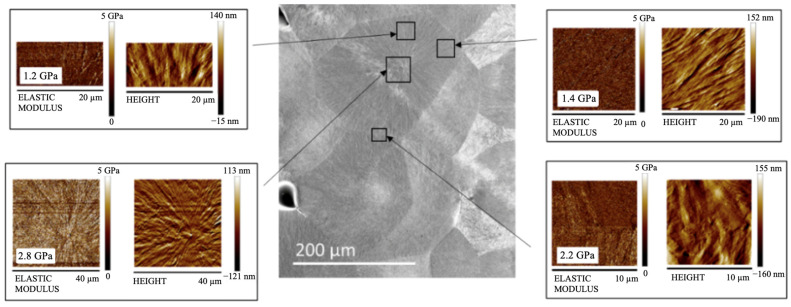
AFM HarmoniX acquisitions performed in several positions on the spherulite for the 160ST case. Each modulus map also reports the average value of the elastic modulus.

DMA mechanical tests revealed that the long annealing step increases the modulus for both adopted mold temperatures; however, HarmoniX acquisitions revealed that part 140ST shows a decrease in the elastic modulus. It has to be noted that the spherulites, which cover most thickness values, are characterized by the distribution of the elastic modulus, with the center showing a higher modulus than the edges. This can be explained by the fact that the center of the spherulite, having developed first, presents a larger density of crystalline lamellae than the edges, which determines higher elastic modulus values [33]. Figure 7 shows the AFM HarmoniX acquisitions, height, and modulus maps for the central position of sample 160ST; in particular, acquisitions were conducted in several positions on the spherulite, as reported in the figure.

Figure 7 confirms that larger values of the elastic modulus can be detected in the spherulite center, consistent with a larger density of crystalline lamellae. In contrast, the edges show lower moduli, 50% lower than in the center. The mechanical characteristic of the spherulites may have altered the distribution along the part thickness (see Figure 6c), which refers to a selected distance from the gate. Such a distance may correspond to the edges of spherulites; thus, a general decrease in the elastic modulus values can be detected. This finding suggests that comparing acquisitions at selected distances from the gate may induce mistakes since this does not account for the distribution of the elastic modulus on the spherulite. In the cases of single annealing, 140FT and 160FT, spherulites are small, and the distributions obtained from AFM are more accurate.

**Figure 8:**
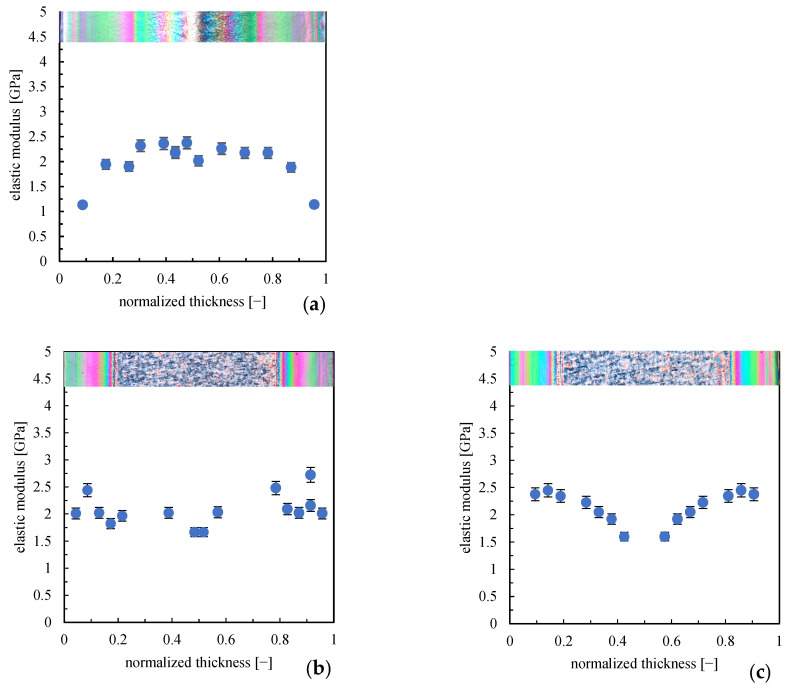
Elastic modulus distribution along the sample thickness in the central position along the flow path obtained by AFM HarmoniX acquisitions for samples (**a**) LT; (**b**) 140FT; (**c**) 160FT.

Figure 8 shows the elastic modulus distributions obtained in the sample center (position along the flow path, see Figure 1).

In the central part of the sample (position along the flow path, see Figure 1), the elastic modulus distribution for sample LT shows maximum values in the core and the smallest values of the modulus toward the sample walls. The highest elastic modulus values observed in the oriented layer in the regions close to the gate are not more detectable downstream from the gate. It has to be noted that the areas close to the gate experience the effect of flow for longer times than the regions at greater distances from the gate, and flow determines the orientation of the polymer chains. The longer the polymer chains experience flow, the higher the orientation (and the structuring level), with a consequent increase in the modulus. Generally, the elastic modulus distribution in the center of the part is more uniform than that obtained at positions close to the gate.

**Figure 9:**
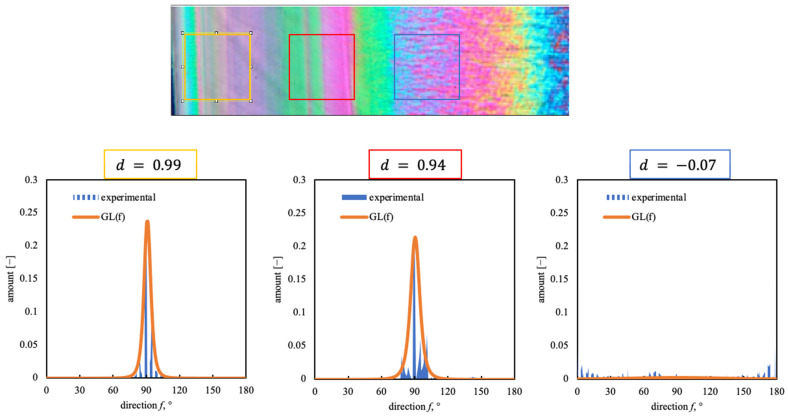
Analysis of Directionality along the sample thickness conducted by Fiji on an optical micrograph (70 × 70 µm windows). Sample LT in gate position (see the sketch in Figure 1).

The flow intensity is a key factor in determining a part’s performance. In particular, flow determines the polymer chain orientation; such an orientation is partially preserved at solidification. The residual orientation along the sample thickness was analyzed in this work. In particular, a method based on optical image analysis has been applied to evaluate the orientation distribution along the thickness, adopting the Directionality plug-in implemented in Fiji. To the best of our knowledge, this method has been applied only to the AFM amplitude error maps, obtaining results almost consistent with those obtained from evaluating Herman’s factor from the WAXS measurement [29]. Figure 9 shows the analysis of Directionality applied to optical micrographs.

**Figure 10:**
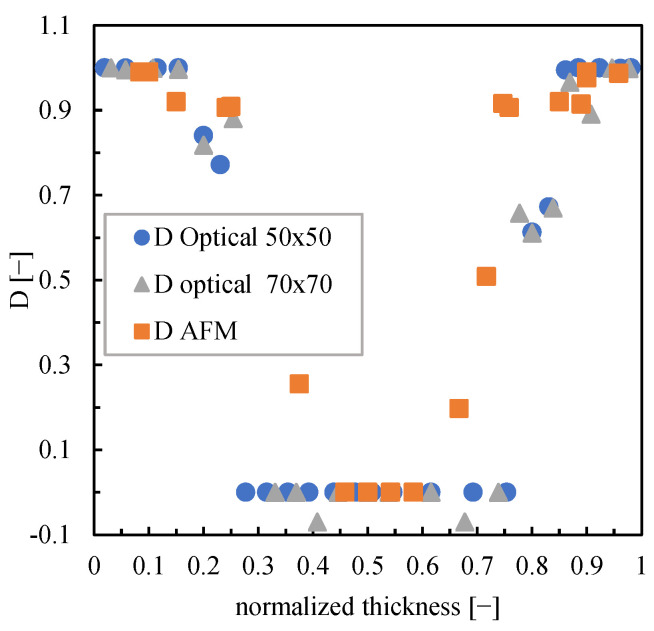
Comparison between parameter D obtained by AFM acquisitions and parameter D obtained by analyzing the optical micrographs for sample LT.

Figure 9 shows that the GL(f) distribution is centered at 90°, the flow direction, for the positions close to the sample surface. The narrowest distributions are obtained close to the sample surface; the GL(f) distribution becomes wider toward the transition region, even if a 90° direction is preserved. The core does not show any direction. Figure 10 shows the comparison between parameter D obtained from the analysis of the optical micrographs for sample LT (considering an investigated area of 50 × 50 µm and 70 × 70 µm) and parameter D obtained from the AFM maps (considering an investigated area of 20 µm).

**Figure 11:**
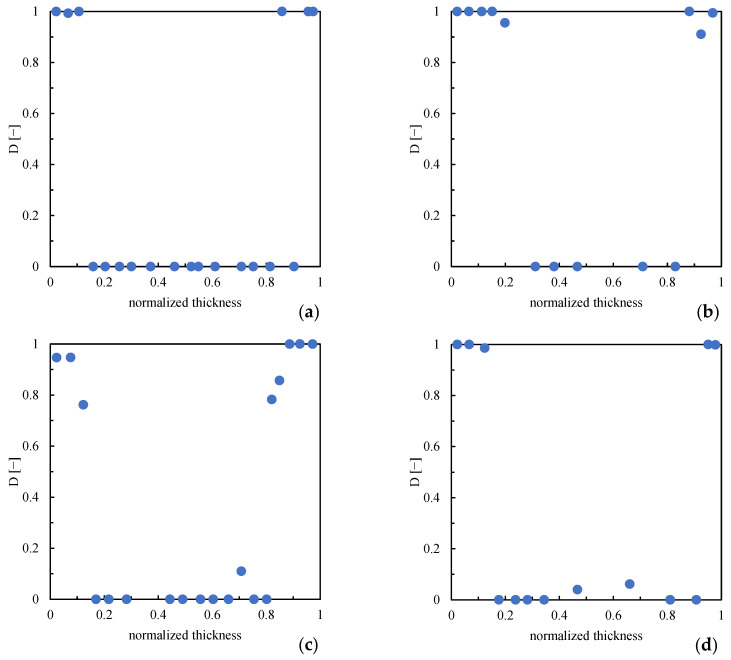
Distribution of parameter D along the sample thickness for samples 140FT (**a**), 140ST (**b**), 160FT (**c**), and 160ST (**d**) at 10 mm downstream from the gate.

The layers close to the surface are characterized by values of parameter D close to 1, which confirms that these regions are highly oriented. Toward the core, parameter D decreases, consistent with the reduction of orientation. The core does not show any orientation (D = 0), whereas, in the transition layer, parameter D  gradually decreases from the high values of the oriented layer toward the lowest values of the core. Parameter D obtained from the analysis of the optical micrographs is consistent with the parameter obtained from the analysis of the AFM maps; this comparison proves the suitability of the directionality analysis applied to the optical micrographs. Figure 11 shows the distribution parameter D along the sample thickness for all molding conditions.

When higher mold temperatures are used, the regions in which parameter D shows values close to 1 decrease in the extension with respect to those observed for sample LT, consistent with the reduction of the oriented layer thickness already observed from optical micrographs. The second annealing has a negligible influence on the thickness of regions with high orientation.

## 5. Discussion

**Figure 12:**
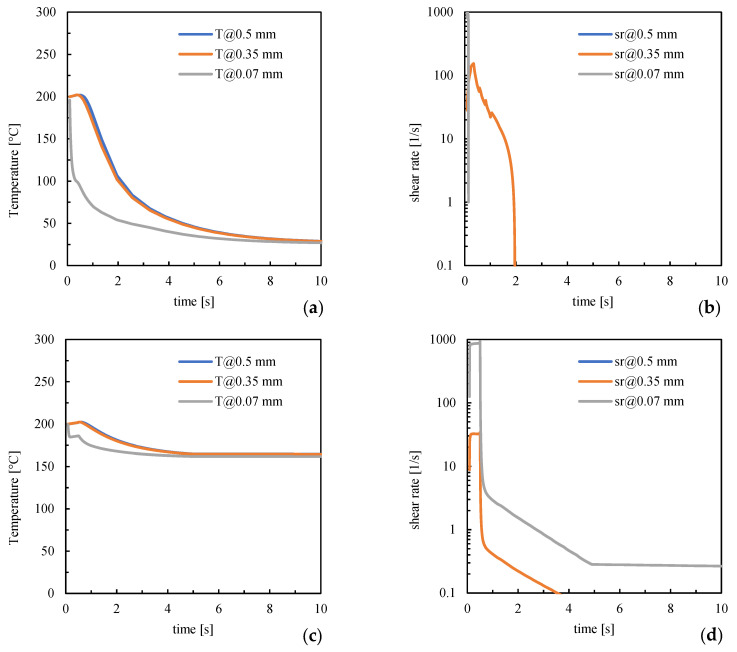
Temperature (**a**–**c**) and shear rate (**b**–**d**) evolutions for cases LT and 160FT at different distances from the sample surface. Simulations refer to the gate position.

Morphology and mechanical performances depend on the crystallization experienced by molecules and the preserved orientation. Previous works demonstrated that crystallization occurs within higher temperature ranges when the mold temperature is high [31]; this would determine the increase in the crystalline degree and elastic modulus [34,35]. Crystallization and orientation depend on the temperature and shear rate experienced by the polymer chains. Figure 12 shows the temperature and shear rate evolutions for cases LT and 160ST obtained from simulations and referring to the gate position (see Figure 1); several distances from the sample surface have been considered.

Figure 12 shows that temperatures are high and close to the injection one in both cases during the early stage of the process (up to 0.5 s). The region adjacent to the surface shows a sudden cooling in the LT case, whereas in the 160ST case, cooling slows due to the mold-settled temperature. Concerning the shear rate, it achieves high values close to the surface; for the LT case, it suddenly decreases due to solidification; for the 160ST case, it remains at high values since high temperatures allow melt flow. Toward the core (0.35 mm distance), the shear rate achieves values smaller than those found close to the surface; however, it retains high values for longer times due to slower cooling. For the LT case, the shear rate sharply decreases at about 2 s due to solidification; for the 160ST case, the flow is allowed for a long time; thus, the shear rate gradually decreases and achieves the smallest values within 4 s. In the core, the shear rate is the smallest (sr = 0 s^−1^) regardless of the molding condition. These results are consistent with the experimental observations related to the orientation distribution: in the LT case, the polymer chains experience stronger flow in the presence of low temperatures and are not allowed to relax; thus, they preserve higher orientation levels at solidification than the 160ST case, showing a smaller extension of the oriented layer.

Irrespective of the conditions adopted during the process, the oriented layers are characterized by parameter D close to 1; thus, the orientation is the maximum. The elastic modulus detected by AFM HarmoniX in the oriented layers is 2.5 ± 1 GPa for all considered cases. The region where the modulus is high decreases in extension consistent with the reduction of parameter D. Such a reduction is gradual for part LT; for such a part, the transition area, where the morphology gradually changes from fibrillar to spherulitic, also shows a gradual decrease in parameter D. Thus, the orientation level determines the value of the elastic modulus.

In the sample core, parameter D shows the lowest values, close to 0, which means that such a layer does not show any orientation. However, the elastic modulus is low only for the parts obtained with a 25 °C mold temperature (LT). In these cases, the fast cooling hinders molecule structuring, and the elastic modulus values mainly depend on the orientation. The elastic modulus for all other cases depends on how the spherulites form. For conditions 140FT and 160FT, spherulites form during cooling in temperature ranges higher than those of case LT. Thus, a smaller number of spherulites forms with dimensions slightly larger than those observed in the LT parts. In these cases, the elastic modulus in the core shows values similar to those found in the oriented layer.

In the cases 140ST and 160ST, the nucleation rate is so slow that only a few spherulites form, with consequent large dimensions. A distribution of the elastic modulus can be detected on each spherulite. Generally, the increase in spherulite dimensions leads to an increase in the elastic modulus.

## 6. Conclusions

This paper explores the influence of in-mold annealing on the mechanical performances of injection molded parts. For this purpose, different annealing cycles were adopted: a first annealing starting soon after the filling, 300 s long; a second annealing was based on the half crystallization time of the adopted iPP (130 °C, 2 h long). Morphological and mechanical characterizations were performed from the macro to the micro scale to reveal the dependence of the performance of the part on the morphological features.

Morphology distribution was characterized by oriented layers close to the sample surface, a transition layer from the end of the oriented layer toward the core, and a grainy core. The extension of such a feature depended on the operating conditions. The analyses proved that the oriented layers formed in the early stages of the process, already during the filling, and the extension depended only on the specified mold temperature. The successive in-mold annealing had a negligible influence on the extension of the oriented layers. Directionality analysis conducted on optical micrographs revealed that the value of orientation, namely parameter D (which was demonstrated to be consistent with Herman’s factor), did not change, regardless of the applied conditions, in the regions close to the sample surface. Consistently, the elastic modulus showed the highest values in these regions.

The results suggested that crystalline structures determine the elastic modulus in the core. The dimension of the features depends on the way the crystallization proceeds. If crystallization mainly occurs during the cooling, small spherulites form. In LT (considered the reference conventional case), spherulites were smaller than in the other cases, with lower elastic modulus. With the increase in mold temperature, spherulites were found to be larger than in the case of LT, with higher modulus. When the second annealing was performed, only a few spherulites with the largest dimensions formed. An elastic modulus distribution was detected on each spherulite, with the highest values in the spherulite center. The parts obtained with two-step annealing were characterized by the highest elastic moduli and proved the efficiency of in-mold annealing in determining the performance of the final parts.

## Figures and Tables

**Table 1:** Operating conditions and test codes adopted during injection molding with in-mold annealing.

Test Code	Mold Temperature, °C	First Annealing		Second Annealing	
		Temperature, °C	Time, s	Temperature, °C	Time, h
LT	25	-	-	-	-
140FT	140	140	300	-	-
140ST	140	140	300	130	2
160FT	160	160	300	-	-
160ST	160	160	300	130	2

**Table 2:** Parameters that describe the temperature dependence of the spherulitic growth rate.

Parameter	Value Regime II	Value Regime III	Unit
U/R	751.6	751.6	K
$G_{0}$	1380	12.5	m/s
$K_{g}$	37,1381	257,408.8	K^2^
$T_{m,0}$	194.54	194.54	°C
$T_{\infty }$	−74.44	−74.44	°C
$b_{6}$	0.0018	0.0018	°C/bar
$a_{n}$	0.155	0.155	(m^3^ K)^−1^
$b_{n}$	16.52	16.52	m^−3^

## Data Availability

Data are contained within the article.
